# Isolated Allergic Conjunctivitis Induced by an Oral Nonsteroidal Anti-inflammatory Drug (NSAID), Confirmed by an Oral Provocation Test: A Case Report

**DOI:** 10.7759/cureus.111704

**Published:** 2026-06-29

**Authors:** Younesse Hidan, Jouhaina Chafi

**Affiliations:** 1 Ophthalmology, Centre Hospitalier Universitaire Ibn Rochd, Casablanca, MAR; 2 Ophthalmology, Hôpital 20 Août 1953, Casablanca, MAR

**Keywords:** allergic conjunctivitis, cox-1 inhibition, drug-induced conjunctivitis, nsaid hypersensitivity, oral provocation test

## Abstract

Nonsteroidal anti-inflammatory drugs (NSAIDs) are a well-known cause of systemic hypersensitivity (urticaria, angioedema, and bronchospasm). Ocular involvement is usually described as merely a component of a systemic reaction. It should be distinguished from the local toxicity of topical ocular NSAIDs.

To our knowledge, very few cases of isolated allergic conjunctivitis induced by oral NSAIDs have been reported. We report the case of a 24-year-old woman with no history of atopy, who had presented for the past 7 months with recurrent bilateral asymmetric allergic conjunctivitis, predominantly affecting the right eye and strictly coinciding with her menstrual cycle. Examination revealed reduced visual acuity due to conjunctival chemosis and tearing, at 20/25 (LogMAR 0.10) in the right eye and 20/22 (LogMAR 0.05) in the left eye. Allergy testing, including prick tests and eosinophil counts, did not identify a relevant causative allergen explaining the clinical presentation. A detailed medication history revealed a temporal correlation with oral ibuprofen used for severe dysmenorrhea, without any accompanying cutaneous, respiratory, or systemic symptoms. Upon discontinuation of ibuprofen and its replacement with paracetamol and codeine, no recurrence was observed for four consecutive months, and an oral provocation test performed in the fifth month was positive, with mild conjunctivitis confirming the diagnosis. We report a case of isolated allergic conjunctivitis induced by oral NSAIDs and confirmed by an oral provocation test, which highlights the importance of a systematic review of medication history in all cases of recurrent conjunctivitis and that oral NSAIDs should be considered in the diagnosis of recurrent conjunctivitis.

## Introduction

Nonsteroidal anti-inflammatory drugs (NSAIDs) are among the most widely used medicines in the world and are a well-recognized cause of hypersensitivity reactions, including anaphylaxis, angioedema, urticaria, and bronchospasm [1]. These reactions are classified by the European Academy of Allergy and Clinical Immunology (EAACI)/European Network on Drug Allergy (ENDA) into five phenotypes and are most often non-immunological, related to COX-1 inhibition rather than to a drug-specific immune response [1]. The ocular involvement described is typically part of a systemic reaction, for example, periorbital edema in the context of urticaria or anaphylaxis [2]. It is therefore important to distinguish between three scenarios: local toxicity of NSAIDs in eye drops, which is a non-systemic surface reaction [3]; periorbital edema as a secondary feature of a systemic reaction; and, as in our case, in which the conjunctiva is the isolated and primary target of a systemic hypersensitivity reaction, without any systemic, cutaneous, or respiratory involvement. To our knowledge, very few cases of isolated allergic conjunctivitis induced by oral NSAIDs have been reported [4]. Such a presentation may easily be overlooked in clinical practice, as recurrent conjunctivitis is usually attributed to seasonal, infectious, or hormonal causes, and a drug-related etiology is rarely considered, especially when symptoms recur in a cyclical pattern. We report the case of a young woman with recurrent, isolated allergic conjunctivitis temporally linked to the use of oral NSAIDs, confirmed by a positive oral provocation test.

## Case presentation

**Figure 1:**
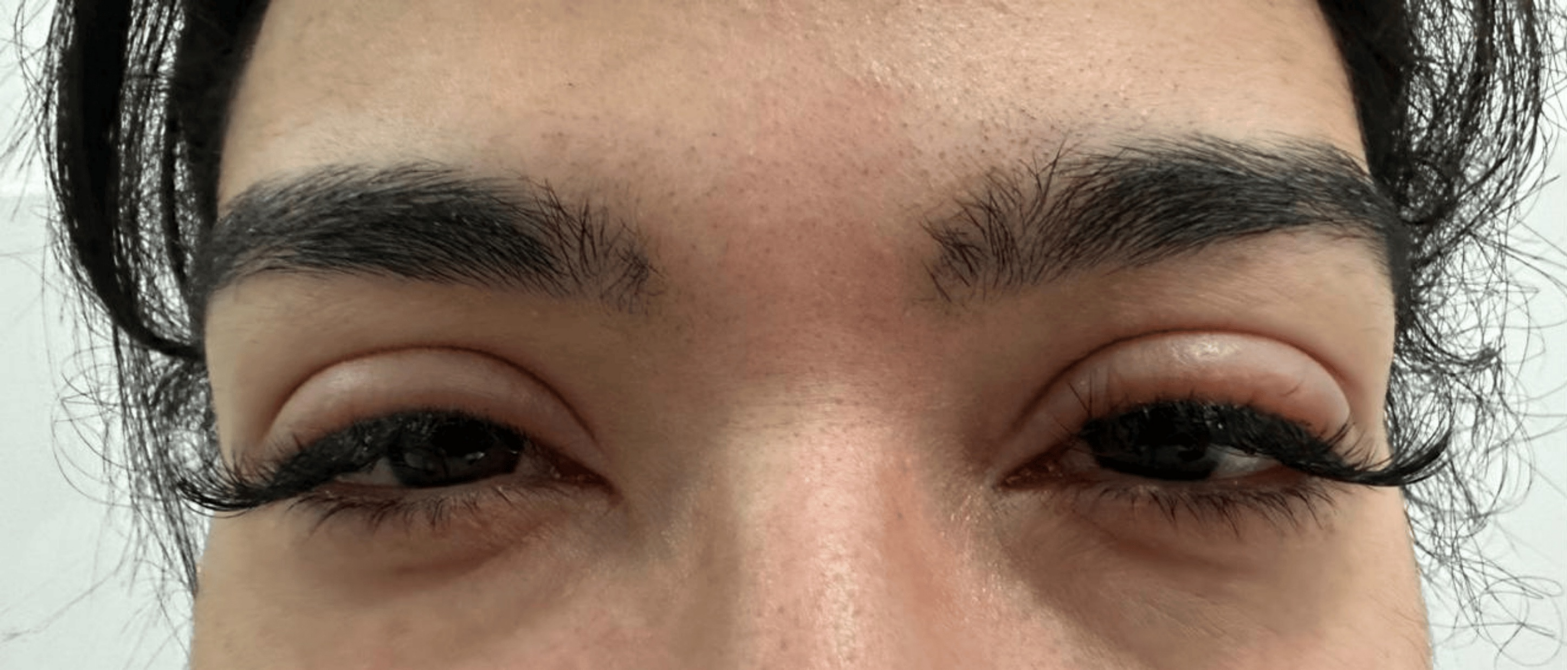
Apparent bilateral asymmetric eyelid swelling due to conjunctival chemosis, predominantly on the right side, following oral ibuprofen Frontal photograph (mirror image) showing apparent bilateral asymmetric eyelid swelling, primarily affecting the right eye, with significant restriction of spontaneous eyelid opening. The swelling corresponds to conjunctival chemosis and infiltration of the palpebral and bulbar conjunctiva rather than to dermal eyelid edema. No rash or changes to the periorbital skin were observed. Photograph taken during an acute episode whilst the patient was receiving oral ibuprofen.

A 24-year-old woman with no significant medical history, including no history of atopy, drug allergies, or eye conditions, presented with a history of recurrent red eye over the past 7 months, accompanied by itching and a slight reduction in visual acuity, which coincided exactly with her menstrual cycle. Each episode resolved spontaneously or following the use of topical corticosteroids within a few days. Ophthalmological examination revealed visual acuity of 20/25 (LogMAR 0.10) in the right eye and 20/22 (LogMAR 0.05) in the left eye, a reduction attributed to chemosis and tearing, with a few papillae visible on eyelid eversion (Figure 1).

The symptoms were more pronounced on the right than on the left; the cornea was clear, with no punctate keratitis, no discharge, and no signs of intraocular inflammation; and the fundus examination was unremarkable. The apparent eyelid edema seen in Figure 1 was secondary to conjunctival infiltration and chemosis of the palpebral and bulbar conjunctiva, which protruded and produced the appearance of eyelid swelling, rather than to dermal or subcutaneous eyelid edema; no cutaneous periorbital signs were present. Overall, the presentation was consistent with bilateral allergic conjunctivitis, though more pronounced on the right. The initial systemic history revealed no symptoms of seasonal allergy, no asthma, no atopic dermatitis, no use of new cosmetics, and no change in living environment or dietary habits. Allergy testing was initiated with skin prick tests and specific IgE tests, which revealed allergies to cockroaches and olive trees, but did not explain the clinical presentation. A detailed history was taken again, with particular emphasis on medication use, where a clear temporal correlation was identified: each episode coincided with the patient's particularly painful menstrual period, for which she had been prescribed oral ibuprofen by her gynecologist. No skin, respiratory or systemic symptoms accompanied the ocular episodes. The diagnosis at this stage was isolated allergic conjunctivitis induced by oral NSAIDs. Management consisted of treating the current episode with topical corticosteroids, a mast cell degranulation inhibitor, and an antihistamine; complete resolution was achieved after 24 hours, and treatment was continued for 15 days. All NSAIDs were discontinued and replaced with paracetamol and codeine for subsequent cycles in the event of significant pain. Thus, four consecutive menstrual cycles without NSAID use were marked by the absence of any ophthalmological symptoms, without any prophylactic or curative anti-allergic therapy. To confirm the diagnosis, an oral ibuprofen challenge test was performed five months after discontinuing the medication. The test was carried out on an outpatient basis under medical supervision: the patient received a single standard daily therapeutic dose of ibuprofen for three consecutive days, replicating the initial exposure conditions rather than following a graded dose-escalation protocol. Because the patient had never presented any systemic, cutaneous, or respiratory manifestation during her previous episodes, the reaction was considered purely local with a low risk of severe systemic reaction, which justified the outpatient setting. Clinical monitoring was carried out, focusing in particular on the onset of ocular and systemic symptoms. The test proved positive, with the onset of mild conjunctivitis, less severe than the usual episodes, confirming the diagnosis. This test was not repeated subsequently following discontinuation of oral ibuprofen. No similar episodes were observed during a two-year follow-up period (Table 1).

**Table 1:** Timeline of recurrent episodes, NSAID exposure, oral provocation test, and follow-up NSAID: nonsteroidal anti-inflammatory drug; VA: visual acuity; OD: right eye; OS: left eye; h: hours

Timepoint	Menstrual cycle	NSAID exposure	Ocular symptoms	Management/Outcome
Episodes 1–7 (over 7 months)	Yes (each episode coincided with menses)	Oral ibuprofen for dysmenorrhea	Recurrent bilateral asymmetric conjunctivitis (right-predominant): chemosis, tearing, itching, reduced visual acuity	Spontaneous resolution or topical corticosteroids within a few days
Index presentation	Yes	Oral ibuprofen	Acute episode; VA 20/25 OD, 20/22 OS; conjunctival chemosis	Topical corticosteroid + mast-cell stabilizer + antihistamine; resolution at 24 h, treatment for 15 days; ibuprofen replaced by paracetamol/codeine
Cycles 1–4 after withdrawal	Yes (4 consecutive cycles)	None (NSAID-free)	None	No symptoms, no anti-allergic therapy
Month 5 - oral provocation test	-	Oral ibuprofen, single standard daily dose × 3 days (outpatient, supervised)	Mild conjunctivitis, less severe than usual episodes	Positive test, confirming diagnosis
Follow-up (2 years)	-	None	None	No recurrence

## Discussion

The EAACI/ENDA classification of hypersensitivity to NSAIDs includes five phenotypes based on the clinical presentation, time of onset, and number of chemical groups involved [1]. Of these phenotypes, NSAID-induced urticaria/angioedema (NIUA) accounts for about one-third of reported cases [2]. The ocular presentation is usually part of a systemic response and includes, most commonly, periorbital edema associated with urticaria or anaphylaxis. Our case is different from the described recognized phenotypes, as the patient presented with isolated conjunctivitis and without cutaneous, respiratory, or systemic manifestations, suggesting a localized and possibly unclassified form of hypersensitivity reaction. We acknowledge that the marked eyelid swelling seen in our patient could raise the possibility of an NIUA/angioedema phenotype; however, this swelling corresponded to conjunctival chemosis and infiltration rather than to dermal eyelid edema, and no cutaneous periorbital signs were present, which argues against angioedema and supports an isolated conjunctival hypersensitivity reaction. NSAID hypersensitivity is usually non-immunological in nature and is associated with the inhibition of cyclooxygenase-1 (COX-1), which redirects the arachidonic acid metabolism to the lipoxygenase pathway, resulting in increased production of leukotriene C4 (LTC4), leukotriene D4 (LTD4), and leukotriene E4 (LTE4) [2]. These inflammatory mediators activate mast cells and release histamine. The conjunctiva is one of the mucosal tissues with the highest density of mast cells [5] and represents a biologically plausible target organ for hypersensitivity reactions. The histamine-induced increases in vascular permeability may explain the clinical manifestations we observed in our patient, such as chemosis, tearing, and conjunctival hyperemia. Systemic reactions would be expected to be symmetric in theory, but the predominance of symptoms in the right eye may be explained by local differences in mast cell density, vascularity, lacrimal drainage, or ocular surface exposure. In our case, causality is strongly supported by several Bradford Hill criteria, including temporal association, reproducibility over 7 consecutive episodes, reversibility after stopping ibuprofen, recurrence after a positive oral challenge test, specificity due to absence of alternative causes, and biological plausibility based on established mechanisms of NSAID hypersensitivity [6]. The combination of a positive challenge and re-challenge constitutes one of the strongest levels of evidence in pharmacovigilance [7]. Systematic differential diagnoses were considered and ruled out. Seasonal allergic conjunctivitis and perennial allergic conjunctivitis were unlikely because symptoms did not show seasonal variation and allergy testing results did not correlate with the clinical presentation. Recurrent infectious conjunctivitis was ruled out by the lack of purulent discharge or other evidence of infection. A hormonal conjunctivitis associated with menstruation was suspected at first, due to the cyclical character of symptoms, but this was ruled out when symptoms abated after ibuprofen withdrawal despite continuing menstrual cycles. Similarly, cyclic dry eye disease was excluded, as the clinical presentation was typical of allergic inflammation rather than tear deficiency. This case also demonstrates a potential diagnostic pitfall of anchoring bias, as the regular monthly recurrence led to a first thought of a hormonal or environmental etiology and a delay in recognition of the iatrogenic cause. Allergic conjunctivitis represents approximately 10-40% of the general population [8,9], with most cases being attributed to airborne allergens. However, isolated conjunctivitis of systemic medications is rarely considered in everyday clinical practice [10]. The recent report of isolated allergic conjunctivitis with oral ketoprofen adds further evidence that the conjunctiva may be the primary or even sole target organ in systemic NSAID hypersensitivity reactions on occasion [4]. Our observation extends the spectrum of adverse reactions associated with NSAIDs and highlights that isolated ocular involvement, although rare, should be considered among the possible clinical manifestations. The positive oral provocation test increases the level of evidence in favor of this diagnosis.

The limitations of this report, however, are that cross-reactivity with other NSAID classes was not tested and that a single non-standardized provocation test was used, which was performed under conditions reproducing the patient's routine exposure and not using a gradual dose-escalation protocol, without a formally documented emergency-precaution protocol given the consistently local and non-systemic nature of the previous episodes.

## Conclusions

We report a rare case of isolated recurring allergic conjunctivitis to oral ibuprofen confirmed by an oral challenge test. This observation broadens the clinical spectrum of hypersensitivity reactions to nonsteroidal anti-inflammatory medications (NSAIDs) and suggests that the conjunctiva may, on rare occasions, be an unusual primary or single target tissue in systemic hypersensitivity. As this is a single case report, oral NSAIDs should be regarded as a rare and possible cause of recurrent allergic conjunctivitis rather than a common or established one, to be considered particularly when symptoms are recurrent and temporally associated with medication use. The complete remission of symptoms upon drug discontinuation and the absence of recurrence during long-term follow-up further reinforce the causal link. This example also demonstrates the necessity of a complete medication history in patients with recurrent conjunctivitis, particularly if the symptoms appear in a cyclical fashion that could initially suggest a hormonal or environmental cause. Therefore, in the differential diagnosis of recurrent allergic conjunctivitis, doctors should consider oral NSAIDs to prevent a delayed diagnosis of rare drug-related presentations.

## References

[1] Kowalski ML, Makowska JS, Blanca M (2011). Hypersensitivity to nonsteroidal anti-inflammatory drugs (NSAIDs) - classification, diagnosis and management: review of the EAACI/ENDA(#) and GA2LEN/HANNA*. Allergy.

[2] Doña I, Pérez-Sánchez N, Eguiluz-Gracia I, Muñoz-Cano R, Bartra J, Torres MJ, Cornejo-García JA (2020). Progress in understanding hypersensitivity reactions to nonsteroidal anti-inflammatory drugs. Allergy.

[3] Bhat C, Rosenberg H, James D (2024). Anti-inflammatoires non stéroïdiens topiques [Article in French]. CMAJ.

[4] Araujo MA (2026). An unusual localized reaction of conjunctivitis with systemic use of ketoprofen: a case report. Case Rep Ophthalmol.

[5] Leonardi A, Castegnaro A, Valerio AL, Lazzarini D (2015). Epidemiology of allergic conjunctivitis: clinical appearance and treatment patterns in a population-based study. Curr Opin Allergy Clin Immunol.

[6] Bradford Hill A (1965). The environment and disease: association or causation?. Proc R Soc Med.

[7] Naranjo CA, Busto U, Sellers EM (1981). A method for estimating the probability of adverse drug reactions. Clin Pharmacol Ther.

[8] Dupuis P, Prokopich CL, Hynes A, Kim H (2020). A contemporary look at allergic conjunctivitis. Allergy Asthma Clin Immunol.

[9] Bjur KA, Lynch RL, Fenta YA, Yoo KH, Jacobson RM, Li X, Juhn YJ (2012). Assessment of the association between atopic conditions and tympanostomy tube placement in children. Allergy Asthma Proc.

[10] Leonardi A, Bozkurt B, Silva D (2025). Drug-induced periocular and ocular surface disorders: an EAACI position paper. Allergy.

